# A cross-sectional evaluation of community pharmacists’ perceptions of intermediate care and medicines management across the healthcare interface

**DOI:** 10.1007/s11096-016-0377-3

**Published:** 2016-09-21

**Authors:** Anna Millar, Carmel Hughes, Maria Devlin, Cristín Ryan

**Affiliations:** 1 School of Pharmacy, Queen’s University Belfast, 97 Lisburn Road, Belfast, BT9 7BL UK; 2 School of Pharmacy, Royal College of Surgeons in Ireland, 111 St. Stephens Green, Dublin 2, Ireland

**Keywords:** Community pharmacy, Healthcare interface, Intermediate care, Medicines management, Questionnaire, United Kingdom

## Abstract

*Background* Despite the importance placed on the concept of the multidisciplinary team in relation to intermediate care (IC), little is known about community pharmacists’ (CPs) involvement. *Objective* To determine CPs’ awareness of and involvement with IC services, perceptions of the transfer of patients’ medication information between healthcare settings and views of the development of a CP–IC service. *Setting* Community pharmacies in Northern Ireland. *Methods* A postal questionnaire, informed by previous qualitative work was developed and piloted. *Main outcome measure* CPs’ awareness of and involvement with IC. *Results* The response rate was 35.3 % (190/539). Under half (47.4 %) of CPs ‘*agreed/strongly agreed*’ that they understood the term ‘intermediate care’. Three quarters of respondents were either not involved or unsure if they were involved with providing services to IC. A small minority (1.2 %) of CPs reported that they received communication regarding medication changes made in hospital or IC settings ‘*all of the time*’. Only 9.5 and 0.5 % of respondents ‘*strongly agreed*’ that communication from hospital and IC, respectively, was sufficiently detailed. In total, 155 (81.6 %) CPs indicated that they would like to have greater involvement with IC services. ‘Current workload’ was ranked as the most important barrier to service development. *Conclusion* It was revealed that CPs had little awareness of, or involvement with, IC. Communication of information relating to patients’ medicines between settings was perceived as insufficient, especially between IC and community pharmacy settings. CPs demonstrated willingness to be involved with IC and services aimed at bridging the communication gap between healthcare settings.

**Electronic supplementary material:**

The online version of this article (doi:10.1007/s11096-016-0377-3) contains supplementary material, which is available to authorized users.

## Impacts on practice

- Intermediate care is an evolving healthcare setting that provides an alternative to hospital for older adults, yet community pharmacists have little awareness of, or involvement with such services.
- Community pharmacists could have a role to play in providing medicines management services to patients in intermediate care, which may improve the currently suboptimal communication of information relating to patients’ medications between hospital, intermediate care, and primary care settings.

## Introduction

Intermediate care (IC) is a care setting that has evolved in response to the ageing population, the increasing pressure faced by acute healthcare services and the resulting need for alternatives to hospital-based care. Whilst various terminologies are used to describe similar care settings globally [1], IC is broadly defined in the United Kingdom (UK) as ‘a range of integrated services to prevent unnecessary hospital admission, promote faster recovery from illness, support timely discharge and maximise independent living’ [2]. Despite the importance placed on the concept of the multidisciplinary team in IC, previous work has highlighted how the pharmacy profession has not been integrated into this care setting [1, 3, 4]. This lack of pharmacy involvement is concerning, given that various aspects of medicines management within the IC setting may be suboptimal [3].

The majority of patients in IC facilities are admitted directly from hospital, and over 70 % of patients return home following discharge from IC [5]. Once home, it can be assumed that the ongoing medicines management of these individuals will be provided by their primary healthcare professionals, including general practitioners (GPs) and community pharmacists (CPs). Patients’ medication regimens are often the subject of change following a period of care in hospital or an IC facility. Sixty percent of patients experience five or more changes to their medicines between admission to and discharge from hospital [6]. It is therefore imperative that information relating to patients’ current medications is communicated effectively to their primary healthcare professionals to ensure continuity of care.

In Northern Ireland (NI), previous qualitative work with CPs has suggested that they have a limited awareness of, and involvement with, IC [3]. Furthermore, it was revealed that CPs frequently experienced challenges relating to the communication of information at the various healthcare interfaces. CPs described often being ‘left out of the loop’, not only in relation to IC, but also the communication of patients’ medication information at the points of transfer between secondary care, IC and primary care. In an attempt to obtain up-to-date information relating to patients’ medications, CPs described how the responsibility fell to them to ‘chase things up.’ Finally, this study’s findings also suggested that CPs could ‘close the loop’ by bridging the gap between healthcare settings, through increased involvement in IC and services targeted at both IC and communication across the healthcare interface [3].

Ineffective communication relating to patients’ medications between healthcare settings may adversely affect patient care [7, 8]. Efforts aimed at improving communication may therefore minimise the potential for medication-related harm. CPs are ideally placed to potentially improve patient-related outcomes by facilitating seamless care when patients are transitioning through the healthcare interfaces [9].

### Aim of the study

The aim of the present study was to further explore and quantify the issues that emerged through the previous qualitative investigation in order to gain a more complete understanding of CPs’ awareness of and involvement in IC facilities in NI and their experiences of the transfer of information at the various existing healthcare interfaces. Additionally, this study aimed to determine CPs’ views of the development of a community pharmacy-IC medicines management service, including their perceived level of confidence in their ability to conduct tasks that may be part of such a service.

### Ethics approval

Ethical approval was obtained from the School of Pharmacy Ethics Committee, Queen’s University Belfast.

## Methods

**Fig. 1:**
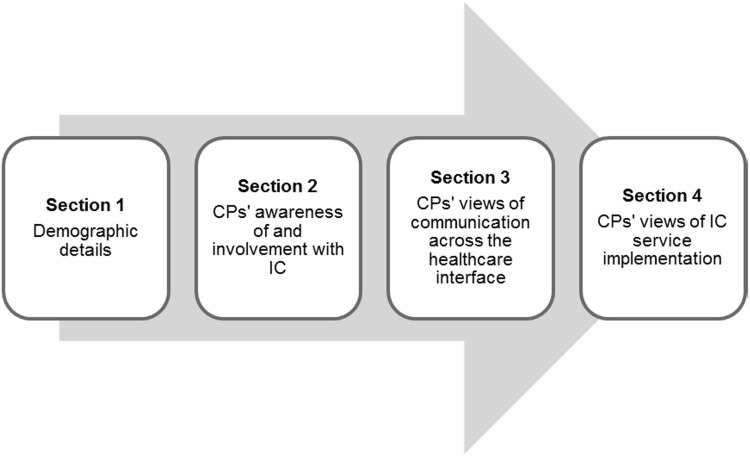
Overview of questionnaire content. Key: *CP* community pharmacist, *IC* intermediate care

This cross-sectional study consisted of an anonymous, self-administered, postal questionnaire. The questionnaire was informed by the findings of previous qualitative work conducted in the area [3] and consisted of four sections (Fig. 1). Questions were largely formatted as either fixed-response options or five-point Likert scales. Two open-ended questions were also included, asking respondents to share their views of communication across the healthcare interface and the development of a community pharmacy-IC service. The questionnaire was piloted with six pharmacists, to assess face and content validity [10].

Community pharmacies were identified through the Pharmaceutical Society of Northern Ireland (PSNI), the regulatory and professional body for pharmacists in NI. The PSNI provide a searchable register of pharmacists and pharmacy premises on their website, however, pharmacists are not linked to the pharmacy within which they practise, nor are their contact details provided. For this reason, the questionnaire was sent by post to every community pharmacy premises in NI (n = 539), addressed to ‘*the pharmacist in charge*’. Questionnaires were posted on two occasions, 3 weeks apart, between January and February 2015. On the first occasion, each pharmacy was sent a pack containing: a letter of invitation, a token incentive (coffee sachet and biscuit), the questionnaire, and a pre-paid return envelope. Informed consent for participation in the study was assumed on receipt of the completed questionnaire.

Responses were entered into SPSS^®^ Version 20.0 (SPSS Inc., Chicago, IL, USA) for analysis. Missing responses were coded as such and omitted from the analysis. A random sample of 10 % of the questionnaires in the electronic database was compared against the paper questionnaires to assess the accuracy of data entry. An error rate of 0.28 % was revealed, and deemed acceptable. Descriptive analyses were conducted to describe the demographics of respondents. Responses to Likert items were analysed by calculating the percentage agreement or disagreement to each statement. Wilcoxon signed-ranked tests were used to explore differences in scores for identical statements relating to different settings (i.e. IC vs. hospital). Scores were calculated based upon the CPs’ responses on a scale of 1–5, where a lower score indicated a greater agreement with a statement and vice versa. Respondents who answered ‘*don’t know*’ were excluded from this analysis. Differences were considered significant if *p* < 0.05.

To determine their perceived level of confidence in their ability (i.e. self-efficacy) to contribute to a IC service, respondents were asked to rate their level of confidence in their ability to provide various aspects of a hypothetical IC service, in line with Bandura’s Social Cognitive Theory [11]. The response format for each of these items was a 10-point self-efficacy scale, where 1 indicated ‘*cannot do at all*’ and 10 indicated ‘*highly certain can do*’ [12].

Responses to open-ended questions were entered into Microsoft Word^®^ (2010) and analysed for emergent themes. Verbatim quotations were used to illustrate identified themes. All respondents were assigned a unique identifier to ensure anonymity.

## Results

### Response rate and demographics

A total of 190 completed questionnaires were returned, corresponding to a response rate of 35.3 %. The demographic details pertaining to the respondents are provided in Table 1. Data from the PSNI relating to all registered pharmacists in NI was obtained to allow for a demographic comparison with the study participants. The information available related to pharmacists working in all sectors, and not solely CPs, who comprised 59 % of those pharmacists registered with the PSNI in 2014.

**Table 1:** Demographic profile of study respondents (n = 190) compared to all pharmacists registered with the PSNI (n = 2003)

	Study respondents n (%)	PSNI n (%)	
Gender			
Male	79 (41.6)	666 (33.3)	
Female	111 (58.4)	1337 (66.7)	
Age (years)		Age	
<25	11 (5.8)	≤25	144 (7.2)
25–34	71 (37.4)	26–35	873 (43.6)
35–44	55 (28.9)	36–45	525 (26.2)
45–54	42 (22.1)	46–55	326 (16.3)
55–64	11 (5.8)	56–65	119 (5.9)
≥65	0 (0.0)	66–70	4 (0.2)
		≥71	12 (0.6)
Years practising			
≤5	42 (22.1)	^a^	
6–11	45 (23.7)		
12–17	35 (18.4)		
18–23	23 (12.1)		
24–29	26 (13.7)		
30–35	14 (7.4)		
≥36	2 (1.1)		
Missing	3 (1.6)		
Type of community pharmacy			
Independent	106 (55.8)	^a^	
Multiple	84 (44.2)		
Location of community pharmacy		^a^	
Urban	84 (44.2)		
Suburban	43 (22.6)		
Rural	62 (32.6)		
Missing	1 (0.5)		
Average number of prescription items dispensed on a weekday			
<50	4 (2.1)	^a^	
50–199	57 (30.0)		
200–400	84 (44.2)		
>400	38 (20.0)		
Missing	7 (3.7)		
Age profile of patients using pharmacy			
Majority <65 years	42 (22.1)	^a^	
Majority ≥65 years	142 (74.7)		
Missing	6 (3.2)		
Additional prescribing qualifications			
None	173 (91.1)	1790 (89.4)	
Supplementary prescriber	7 (3.7)	14 (0.7)	
Independent prescriber	10 (5.3)	199 (9.9)	
Currently using prescribing qualification (of those qualified)			
Yes	8 (29.6)	^a^	
No	19 (70.4)		

^a^Data unavailable from the Pharmaceutical Society of Northern Ireland

### Awareness of and involvement with intermediate care

Less than half (90; 47.4 %) of CPs either ‘*agreed*’ or ‘*strongly agreed*’ that they understood what was meant by the term ‘*intermediate care*’, and fewer (70; 36.8 %) ‘*agreed*’ or ‘*strongly agreed*’ that they were aware of the IC facilities in their area. Despite these findings, 152 (80.0 %) CPs ‘*agreed*’ or ‘*strongly agreed*’ that CPs (generally) should have greater involvement with IC services. A similar number (155; 81.6 %) ‘*agreed*’ or ‘*strongly agreed*’ that they (personally) would like to be more involved with IC services (Table 2).

**Table 2:** CPs’ agreement with statements regarding awareness of and involvement with intermediate care

Statement	SA n (%)	A n (%)	NAD n (%)	D n (%)	SD n (%)	M n (%)
I understand what is meant by the term ‘*intermediate care*’	11 (5.8)	79 (41.6)	34 (17.9)	53 (27.9)	12 (6.3)	1 (0.5)
I am aware of the intermediate care facilities in my local area	9 (4.7)	61 (32.1)	28 (14.7)	78 (41.1)	12 (6.3)	2 (1.1)
I think community pharmacists should have greater involvement with intermediate care facilities/services	64 (33.7)	88 (46.3)	29 (15.3)	8 (4.2)	0 (0.0)	1 (0.5)
I would like to have greater involvement with intermediate care facilities/services	63 (33.2)	92 (48.4)	26 (13.7)	8 (4.2)	0 (0.0)	1 (0.5)

*SA* strongly agree, *A* agree, *NAD* neither agree nor disagree, *D* disagree, *SD* strongly disagree, *M* missing

The majority of CPs (142; 74.7 %) were either not providing any services to IC facilities, or were unsure if they were providing services. Of the 46 (24.2 %) CPs who reported that they provided services to IC facilities, the most frequently provided service was the dispensing of medication to patients who would regularly use their pharmacy and were subsequently admitted to IC (41; 89.1 %).

### Communication across the healthcare interface

**Fig. 2:**
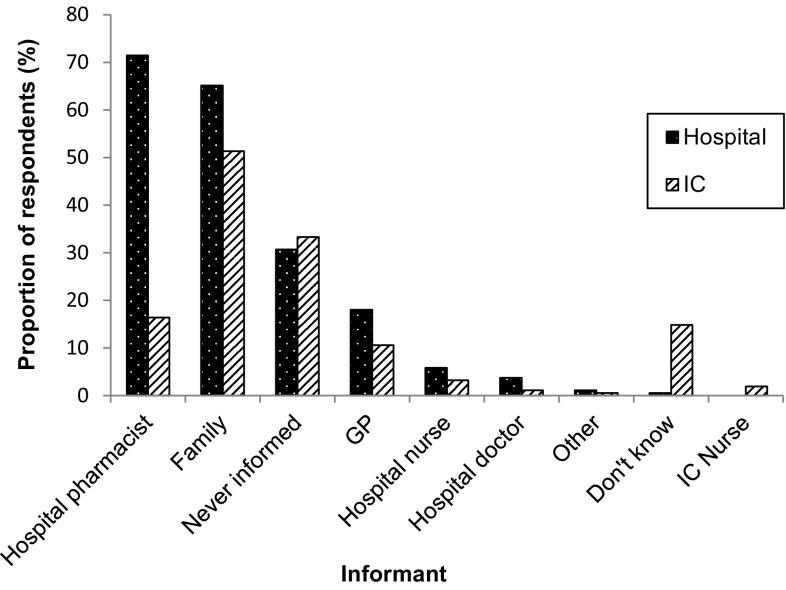
Main informants for the CP when a patient who used their pharmacy was admitted to hospital or an IC facility. Key: *GP* general practitioner, *IC* Intermediate care

CPs were asked to indicate who would typically inform them when a patient who regularly used their pharmacy was admitted to IC or hospital. Figure 2 shows the categories of informants and the proportion of CPs who indicated that these individuals would typically notify them of a patient’s admission.

Approximately one-third of respondents reported that they were ‘*never informed*’ when a patient who used their pharmacy was admitted to either hospital or IC. CPs described ‘*other*’ informants as including ‘home-help’, nursing home staff and pharmacy delivery drivers.

CPs were asked, in general, how frequently they would be informed of changes made to patients’ medicines at discharge from hospital and IC. Seventy-five (39.5 %) CPs indicated that changes in the dose or frequency of medicines were communicated from hospital ‘*most of the time*’. Similarly, 70 (36.8 %) and 63 (33.2 %) respondents reported that new medicines and stopped medicines, respectively, were communicated *‘most of the time’*. On average, 36.5 % of CPs reported that changes to patients’ medication regimens (of any type) made in hospital were communicated ‘*most of the time*’. Considering changes made in the IC setting, the corresponding value was less than half that relating to hospitals (17.4 %). Combining both hospital and IC, only 1.2 % of CPs reported that they received communication regarding medication changes made ‘*all of the time*’.

CPs were also asked the methods by which patients’ medication information was transferred to them at discharge from hospital or IC, in those instances when information was indeed communicated. Respondents could select more than one option. A telephone call was the most frequently reported, as 156 (82.1 %) and 54 (28.4 %) CPs indicated that they received communication via this method from both hospital and IC, respectively. Email was the least popular as only 17 (8.9 %) and one (0.5 %) CP(s) indicated that they receive communications via this method from hospital and IC, respectively.

CPs were asked to indicate their views on additional aspects of communication across the healthcare interface. One hundred and six (55.7 %) respondents ‘*agreed*’ or ‘*strongly agreed*’ that communication between GP surgeries and their community pharmacy was good. However, only 26 (13.7 %) ‘*agreed*’ or ‘*strongly agreed*’ that communication between IC facilities and their community pharmacy was good. Less than one in ten (9.5 %) CPs ‘*strongly agreed*’ with the statement: ‘At patient discharge, the level of detail provided in medication communication information from hospital is sufficient for my needs as a community pharmacist.’ For IC, this figure fell to 0.5 % (Table 3).

**Table 3:** CPs’ agreement with statements regarding communication between community pharmacy and various healthcare interfaces

Statement	SA n (%)	A n (%)	NAD n (%)	D n (%)	SD n (%)	DK n (%)	M n (%)	Score (median; IQR)	Wilcoxon signed-ranks test (two tailed)
Communication is good between my pharmacy and									
GPs	17 (8.9)	89 (46.8)	31 (16.3)	36 (18.9)	17 (8.9)	–	–	–	–
IC	1 (0.5)	25 (13.2)	53 (27.9)	48 (25.3)	24 (12.6)	39 (20.5)	–	3.0; 3.0–4.0	Z = −6.67, p < 0.01
Hospital	6 (3.2)	87 (45.8)	48 (25.3)	35 (18.4)	13 (6.8)	1 (0.5)	–	2.5; 2.0–3.0	
Sufficient information is communicated to CP at discharge from									
Hospital	18 (9.5)	88 (46.3)	24 (12.6)	35 (18.4)	24 (12.6)	–	1 (0.5)	2.0; 2.0–4.0	Z = −7.02, p < 0.001
IC	1 (0.5)	25 (13.2)	48 (25.3)	42 (22.1)	28 (14.7)	45 (23.7)	1 (0.5)	3.0; 3.0–4.0	
I often have to contact GP to obtain medication information on patients’ medication after discharge from									
Hospital	78 (41.1)	71 (37.4)	19 (10.0)	19 (10.0)	2 (1.1)	–	1 (0.5)	2.0; 1.0–2.0	Z = −1.57, p = 0.116
IC	63 (33.2)	58 (30.5)	24 (12.6)	4 (2.1)	1 (0.5)	38 (20.0)	2 (1.1)	2.0; 1.0–2.0	
Information contained in discharge summaries is clearly presented from									
Hospital	19 (10.0)	109 (57.4)	32 (16.8)	22 (11.6)	2 (1.1)	4 (2.1)	2 (1.1)	2.0; 2.0–3.0	Z = 6.29, p < 0.01
IC	–	35 (18.4)	63 (33.2)	21 (11.1)	6 (3.2)	63 (33.2)	2 (1.1)	3.0; 2.0–3.0	
Information relating to patients’ medications following discharge is communicated to me in a timely manner									
Hospital	4 (2.1)	81 (42.6)	46 (24.2)	41 (21.6)	15 (7.9)	2 (1.1)	1 (0.5)	3.0; 2.0–4.0	Z = −4.96, p < 0.001
IC	1 (0.5)	22 (11.6)	63 (33.2)	35 (18.4)	15 (7.9)	52 (27.4)	2 (1.1)	3.0; 3.0–4.0	
I would like to receive more information on patients’ medications at discharge from									
Hospital	86 (45.3)	58 (30.5)	21 (11.1)	22 (11.6)	2 (1.1)	–	1 (0.5)	2.0; 1.0–2.0	Z = −4.52, p < 0.001
IC	97 (51.1)	55 (28.9)	18 (9.5)	2 (1.1)	–	17 (8.9)	1 (0.5)	1.0; 1.0–2.0	
It’s important for me to know a patient’s diagnosis/reason for admission to									
Hospital	45 (23.7)	95 (50.0)	37 (19.5)	11 (5.8)	–	–	2 (1.1)	2.0; 1.0–3.0	Z = −2.53, p < 0.05
IC	44 (23.2)	81 (42.6)	45 (23.7)	9 (4.7)	–	10 (5.3)	1 (0.5)	2.0; 2.0–3.0	
It’s important for me to know the reason(s) for changes made to patients’ medication in									
Hospital	72 (37.9)	91 (47.9)	19 (10.0)	5 (2.6)	1 (0.5)	–	2 (1.1)	2.0; 1.0–2.0	Z = −2.24, p < 0.05
IC	69 (36.3)	85 (44.7)	20 (10.5)	5 (2.6)	1 (0.5)	9 (4.7)	1 (0.5)	2.0; 1.0–2.0	
I think CPs should have access to patients’ medical records in community pharmacies									
	100 (52.6)	58 (30.5)	19 (10.0)	8 (4.2)	3 (1.6)	1 (0.5)	1 (0.5)	–	–
I think patients should be registered with one community pharmacy to ensure continuity of care at healthcare interfaces									
	77 (40.5)	60 (31.6)	31 (16.3)	15 (7.9)	3 (1.6)	3 (1.6)	1 (0.5)	–	–

*SA* strongly agree, *A* agree, *NAD* neither agree nor disagree, *D* disagree, *SD* strongly disagree, *DK* don’t know, *M* missing, *IQR* interquartile range, *GP* general practitioner, *IC* intermediate care, *CP* community pharmacist

For both hospital and IC settings, the vast majority of CPs indicated that they often had to contact a GP to obtain information relating to patients’ medication after discharge. Only 19 (10.0 %) respondents ‘*strongly agreed*’ that information contained in discharge summaries from hospitals was clearly presented. Only four (2.1 %) and one (0.5 %) respondents ‘*strongly agreed*’ that information from hospitals and IC, respectively, was communicated to them in a timely manner. The vast majority (144; 75.8 % and 152; 80.0 %) either ‘*strongly agreed*’ or ‘*agreed*’ that they would like to receive more information relating to patients’ medications at discharge from hospital and IC, respectively.

Excluding those who answered ‘*don’t know*’, a total of 150 (78.9 %) respondents’ views were compared in relation to the statements: ‘Overall I think the communication between IC facilities and my community pharmacy is good’, and ‘Overall, I think the communication between hospitals and my community pharmacy is good’. Significantly more CPs were in agreement with the statement in relation to hospitals (median score 2.5; interquartile range 2.0–3.0) compared with IC facilities (median score 3.0; interquartile range 3.0–4.0), z = −6.67, *p* < 0.001.

**Fig. 3:**
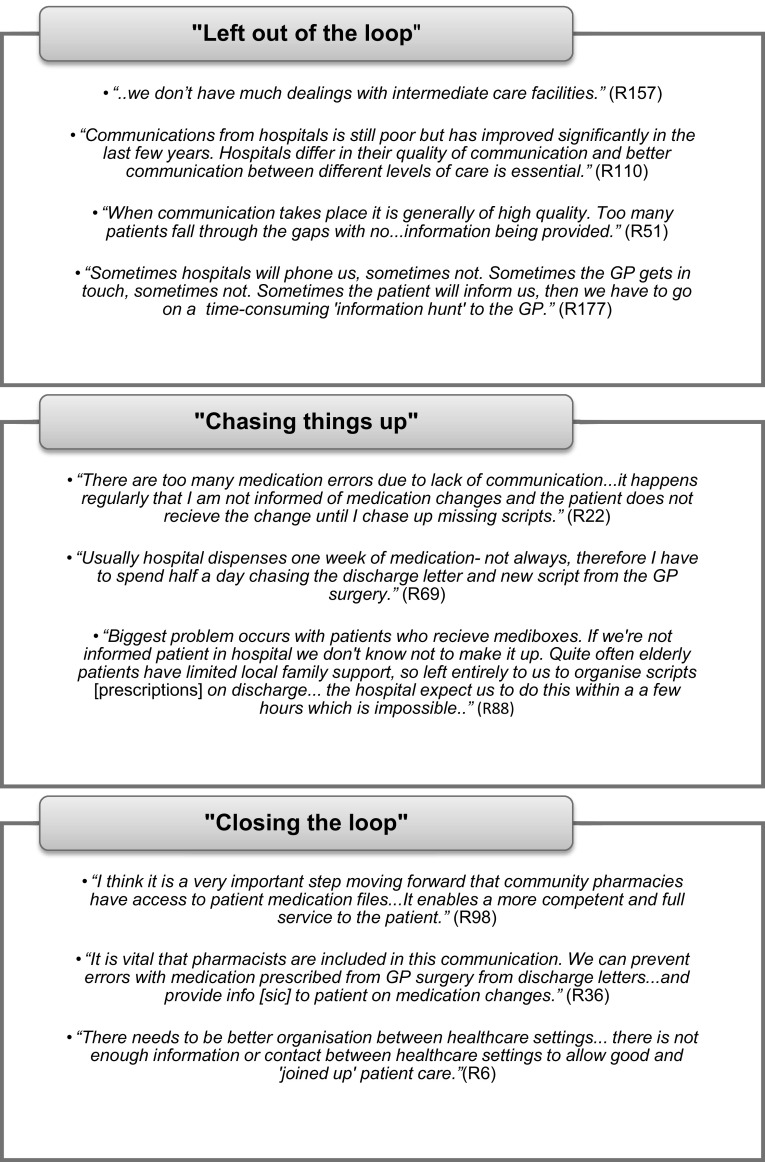
CPs’ views of communication between the various healthcare interfaces

Respondents were asked if they had any further comments on communication across the healthcare interface. Three themes emerged from the data: ‘left out of the loop’, ‘chasing things up’ and ‘closing the loop’. Figure 3 highlights these themes with supporting quotations from respondents.

### Community pharmacy: intermediate care service implementation

When asked about their confidence in conducting specific tasks with patients and/or staff in IC facilities, CPs were generally highly confident in their ability to conduct all those suggested, as evident from the mean self-efficacy scores for each item (Table 4), with the possible score range being 1-10, where 1 indicated ‘*cannot do at all*’ and 10 indicated ‘*highly certain can do*’ [12].

**Table 4:** CPs’ self-efficacy scores for a range of IC service tasks

Task	Self-efficacy mean score (±SD)
Counseling IC patients on their medicines	8.68 (±1.59)
Providing education to IC staff	8.40 (±1.78)
Reconciling IC patients’ medicines	8.55 (±1.65)
Providing prescribing advice/make recommendations to prescribers on appropriateness of IC patients’ medicines	7.65 (±2.28)

CPs were then provided with a list of potential barriers to the development of an IC service and asked to rate each in order of importance to them. ‘*Current workload*’ was rated by the majority (58; 40.0 %) of respondents as the most important barrier. Despite comments suggesting that such a service would be conceptually viable, CPs reported that several barriers would need to be addressed prior to the implementation of such services. Reimbursement of services and the additional staff needed in order to provide such services were highlighted frequently by respondents. Nevertheless, comments received from respondents indicated that CPs viewed themselves as being ideally placed to being involved with IC services and/or services that would facilitate patients’ transitions across the healthcare interface:

Community pharmacy is ideally placed to deal with issues in intermediate care and should have an important role to play. (R55)

[CPs] are extremely competent in providing advice rather than just dispensing. We have a fountain of knowledge yet rarely get to use it. (R73)

[CPs] are ideally placed to follow up on discharge medication reviews and prevent readmission due to medication errors. (R170)

## Discussion

The study highlighted a low awareness of and involvement with IC services amongst CPs in NI. This finding is unsurprising given the confusion surrounding the terminology used to describe IC [1]. Despite its presence within the UK for over a decade, IC does not relate to a single healthcare service or setting [13]. However, a majority of CPs reported willingness for the profession to have greater involvement with IC.

The questionnaire generated a response rate of 35.3 %. Whilst not optimal, this response rate is typical of postal questionnaires administered to the sample population [14–17] and the demographic profile of the respondents was not dissimilar to that provided by the PSNI.

The dispensing of medicines to patients in IC accounted for the majority of ‘services’ provided by CPs to IC facilities. In recent times, pharmacists have adopted a variety of enhanced roles, including prescribing, which reflect their expertise surrounding medicines. Whilst not widely implemented in IC, pharmacist prescribing has become an increasingly commonplace practice in both primary and secondary care settings [18, 19]. Notably, it has been shown that patients generally regard pharmacist prescribing as an acceptable alternative to medical prescribing [20, 21]. This study suggests that CPs are keen to expand their professional boundaries, however, it remains the case that the majority of those who have acquired prescribing qualifications are currently not using them, perhaps due to a lack of opportunities or lack of access to clinical information in the community pharmacy setting necessary to facilitate a prescribing role.

CPs viewed communication across the various healthcare interfaces to be deficient. This finding reiterates that reported in the previous qualitative study [3], where it was described how CPs were not routinely informed when patients were admitted into hospital or IC. This issue is not unique to NI [21]. Irrespective of the setting, only a minority of CPs reported that they were informed of changes to patients’ medication regimens ‘*all of the time*’ at discharge. This poses a risk to patients as communication breakdown is a leading cause of adverse events at transitions of care [23]. Furthermore, this study provided additional evidence of CPs ‘chasing things up’ with GPs as a means of accessing information. This ad-hoc method is both inefficient and potentially hazardous. In recognition of this, there have been calls for pharmacists to have access to patients’ records [24]. Additionally, electronic communication of discharge letters has been shown to facilitate the timely transfer of information between settings [8, 25].

Both the content and level of detail contained in communications regarding patients’ medicines was found to be important to CPs. A study by Munday et al. [26] also reported that the majority of CPs considered it necessary to be informed of the reasons underpinning medication changes, yet few received such information. Urban et al. [27] also reported that the provision of information to community pharmacies from hospitals regarding medication was inconsistent and lacking in quality. By routinely providing such level of detail, via a discharge summary, CPs will be able to ascertain whether apparent changes made to medicines are intentional, therefore negating the need to ‘chase things up’.

CPs considered themselves ideally placed and capable of providing services to IC patients and/or staff, as evidenced by the high levels of reported self-efficacy. More than 10 years ago, the Royal Pharmaceutical Society of Great Britain outlined how pharmacists could contribute to IC services [28]. Whilst there remains a lack of involvement from the community pharmacy sector of the profession, there have been emerging examples of innovative models of clinical pharmacist-led care pathways under development in England [29] and NI [30]. This study suggests that the most pertinent barrier to CP involvement is the existing workload that CPs currently face. Further research should therefore aim to determine whether CP–IC services are feasible and have the ability to improve patient outcomes by facilitating seamless care across the healthcare interfaces.

### Strengths and limitations

This study has provided quantitative evidence which further supports the findings of the previous qualitative research [3, 4]. Whilst effortswere taken to optimise the response rate, the low response rate achieved may limit the generalisability of the findings. A poor awareness of IC among CPs may itself have hindered the response rate. The potential for differences in the respondent sample should be acknowledged when interpreting the data, as should the possibility of social desirability bias.

## Conclusion

This study supports the findings of the previous qualitative work whereby CPs in NI demonstrated a lack of awareness of IC and the majority had no involvement with local IC services. In the study described here, the communication of information relating to patients’ medications between healthcare settings was reported to be suboptimal both in quantity and quality, particularly in relation to communication between IC settings and community pharmacies. CPs would like to have greater involvement with IC services and services aimed at bridging the communication gap between the healthcare interfaces. However, important barriers exist that would need to be addressed prior to the development of any service.

## Electronic supplementary material

Below is the link to the electronic supplementary material.

Supplementary material 1 (DOCX 30 kb)
