# Supplementary material for: A cross-sectional evaluation of community pharmacists’ perceptions of intermediate care and medicines management across the healthcare interface
Source: Int J Clin Pharm. 2016 Sep 21;38(6):1380–9. doi: 10.1007/s11096-016-0377-3 (PMC5124038; doi:10.1007/s11096-016-0377-3)

**Medicines management across the healthcare interface**

**HOW TO FILL IN THIS QUESTIONNAIRE**

This questionnaire should take approximately **15 minutes** to complete.

All the information gathered will be anonymous and cannot be linked to you as an individual.

There are no right or wrong answers and all answers are useful.

We are interested in your own personal views, not what you think we want to hear.

| **SECTION 1: DEMOGRAPHIC INFORMATION** |
| --- |

***This section of the questionnaire is concerned with gathering some details about you and where you work.***

**Please select the option that best applies to you:**

**1. Type of community pharmacy you are currently working in:** Multiple □ Independent □

**2. Location of the pharmacy you are currently working in**: Urban □ Suburban □ Rural □

**3. How many items (on average) would this pharmacy dispense on a typical weekday?**

| <50 □ | 50-199 □ | 200-400 □ | >400 □ |
| --- | --- | --- | --- |

**4. Thinking about the patients who use your pharmacy:**

| The majority of patients are younger people (<65 years old) □ |
| --- |
| The majority of patients are older people (>65 years old) □ |

**5. Your gender:** Male □ Female □

**6. Your age (years):**

| <25 □ | 25-34 □ | 35-44 □ | 45-54 □ | 55-64 □ | >65 □ |
| --- | --- | --- | --- | --- | --- |

**7. For how many years have you been practising as a pharmacist?­­­­­­­_____________**

**8A. Are you currently a supplementary or independent prescriber?**

Yes (supplementary) □ Yes (independent) □ No □

***(If you answered yes to Q.8A)***

**8B. Are you currently using this qualification?** Yes □ No □

**9. Do you have any (other) postgraduate qualifications?** Yes □ No □

***If yes,*** please detail below:

----------------------------------------------------------------------------------------------------------------------------------------------------------------------------------------------------------------------------------------------------------------------------

| **SECTION 2: AWARENESS OF INTERMEDIATE CARE** |
| --- |

***The following questions are about intermediate care facilities in Northern Ireland.***

**10. Please rate your level of agreement with the following statement by placing a tick (✓) in the appropriate box:**

|  | **Strongly agree** | **Agree** | **Neither agree nor disagree** | **Disagree** | **Strongly disagree** |
| --- | --- | --- | --- | --- | --- |
| I understand what is meant by the term *‘intermediate care’* | □ | □ | □ | □ | □ |

| **Please consider the following definition of *‘intermediate care’*:** |
| --- |
| *Intermediate care:*   - Describes a range of integrated services to prevent unnecessary hospital admission, promote faster recovery from illness, support timely discharge and maximise independent living - Is provided in facilities including community hospitals, nursing homes and residential care homes - Should be targeted at people who would otherwise face: - Inappropriate admission to acute in-patient care; - Long-term residential/nursing home care; - Should be time-limited, usually no longer than six weeks.   Adapted from: DHSSPS Definition of Intermediate Care. *Elderly and Community Care Unit Circular 2/2005* |

**11. Please indicate your level of agreement with the following statements by placing a tick (✓) in the appropriate box:**

|  | **Strongly Agree** | **Agree** | **Neither agree or disagree** | **Disagree** | **Strongly disagree** |
| --- | --- | --- | --- | --- | --- |
| I am aware of the intermediate care facilities in my local area | □ | □ | □ | □ | □ |
| I think community pharmacists should have greater involvement with intermediate care facilities/services | □ | □ | □ | □ | □ |
| I would like to have greater involvement with intermediate care facilities/services | □ | □ | □ | □ | □ |

**12. I am currently involved in providing services (e.g. dispensing service or other) to intermediate care facilities:**

| Yes □ No □ Not sure □ |
| --- |
| If you answered *‘Yes’*, please go to Q.13 |
| If you answered *‘No’* or *‘Not sure’*, please go to Section 3 (Q.14). |

**13. *If yes*, please indicate what types of services you/your pharmacy provides to intermediate care facilities *(tick all that apply).***

| Dispensing of medication to patients who normally use this pharmacy □ |
| --- |
| Dispensing of medication to patients who don’t normally use this pharmacy □ |
| Providing advice to the staff in the facility regarding medicines □ |
| Other □ *(please specify):* |

---------------------------------------------------------------------------------------------------------------------------------------------------------------------------------------------------------------------------------------------------------------------------------------------------------------------------------------------------------------------------------------------------------------------------------------------------------

| **SECTION 3: COMMUNICATION ACROSS THE HEALTHCARE INTERFACE** |
| --- |

***The following questions focus on your experiences of the communication of patients’ medication information, when patients transfer between healthcare settings (i.e. communication across the healthcare interface). This could be, for instance, when a patient is admitted to or discharged from a hospital or an intermediate care facility.***

**14. In general, if a patient who uses your pharmacy is admitted to hospital, who would inform you of this? *(Tick all that apply)***

| Hospital nurse □ | Patient’s GP/surgery □ |
| --- | --- |
| Hospital pharmacist □ | Other □ *(please specify):_______________________* |
| Hospital doctor □ | Never informed □ |
| Patient’s family □ | Don’t know □ |

**15. In general, if a patient who uses your pharmacy is admitted to an intermediate care facility, who would inform you of this? *(Tick all that apply)***

| Nurse/care staff in intermediate care facility □ | Patient’s family □ |
| --- | --- |
| Hospital nurse □ | Never informed □ |
| Hospital doctor □ | Don’t know □ |
| Hospital pharmacist □ | Other □  *(please specify):_______________________* |
| Patient’s GP/surgery □ |  |
|  |  |

**When patients are admitted to a hospital or intermediate care facility, their medication regimens are often subject to change. Bearing this in mind, please consider the following statements and indicate your response by placing a tick (✓) in the appropriate box.**

**16. Please indicate your experience regarding a patient’s stay in hospital:**

|  | **All of the time** | **Most of the time** | **Some of the time** | **None of the time** |
| --- | --- | --- | --- | --- |
| Following a stay in hospital, changes made to medications (*e.g.* increased strength, different formulation) are explicitly communicated to me… | □ | □ | □ | □ |
| Following a stay in hospital, decisions to start a new medication are explicitly communicated to me… | □ | □ | □ | □ |
| Following a stay in hospital, decisions to stop a medication are explicitly communicated to me… | □ | □ | □ | □ |

**17. Please indicate your experience regarding a patient’s stay in intermediate care:**

|  | **All of the time** | **Most of the time** | **Some of the time** | **None of the time** | **Don’t know** |
| --- | --- | --- | --- | --- | --- |
| Following a stay in intermediate care, changes made to medication (*e.g.* increased strength, different formulation) are explicitly communicated to me… | □ | □ | □ | □ | □ |
| Following a stay in intermediate care, decisions made to start a new medication are explicitly communicated to me… | □ | □ | □ | □ | □ |
| Following a stay in intermediate care, decisions made to stop a medication are explicitly communicated to me… | □ | □ | □ | □ | □ |

**18. Typically, at the point of discharge from hospital or intermediate care facilities, patients’ medication information is communicated to me via: *(Tick all that apply)***

| **Method of communication** | **Hospital** | **Intermediate care** |
| --- | --- | --- |
| Telephone call | **□** | **□** |
| Fax (*e.g.* discharge letter/summary | **□** | **□** |
| E-mail (*e.g.* discharge letter/summary) | **□** | **□** |
| Patient/family (*e.g.* Medication Record Sheet) | **□** | **□** |
| Other method (please specify): __________________________ | **□** | **□** |
| No communication | **□** | **□** |
| Don’t know | **□** | **□** |

**19.** **Please indicate the extent to which you agree or disagree with the following statements by placing a tick (✓) in the appropriate box:**

|  | **Strongly agree** | **Agree** | **Neither agree or disagree** | **Disagree** | **Strongly disagree** | **Don’t know** |
| --- | --- | --- | --- | --- | --- | --- |
| Overall, I think the communication between GP surgeries and my community pharmacy is good | □ | □ | □ | □ | □ | □ |
| Overall, I think the communication between intermediate care facilities and my community pharmacy is good | □ | □ | □ | □ | □ | □ |
| Overall, I think the communication between hospitals and my community pharmacy is good | □ | □ | □ | □ | □ | □ |
| At patient discharge, the level of detail provided in medication communication information from hospital is sufficient for my needs as a community pharmacist | □ | □ | □ | □ | □ | □ |
| At patient discharge, the level of detail provided in medication communication information from intermediate care is sufficient for my needs as a community pharmacist | □ | □ | □ | □ | □ | □ |
| I often find that I have to contact GP surgeries in order to obtain information relating to patients’ medications following discharge from hospital | □ | □ | □ | □ | □ | □ |
| I often find that I have to contact GP surgeries in order to obtain information relating to patients’ medications following discharge from intermediate care | □ | □ | □ | □ | □ | □ |
|  | **Strongly agree** | **Agree** | **Neither agree or disagree** | **Disagree** | **Strongly disagree** | **Don’t know** |
| Information contained in hospital discharge summaries/discharge prescriptions is clearly presented | □ | □ | □ | □ | □ | □ |
| Information contained in intermediate care discharge summaries/discharge prescriptions is clearly presented | □ | □ | □ | □ | □ | □ |
| Information relating to patients’ medications following discharge from hospital is communicated to me in a timely manner | □ | □ | □ | □ | □ | □ |
| Information relating to patients’ medications following discharge from intermediate care is communicated to me in a timely manner | □ | □ | □ | □ | □ | □ |
| I would like to receive more information on patients’ medications at discharge from hospital | □ | □ | □ | □ | □ | □ |
| I would like to receive more information on patients’ medications at discharge from intermediate care | □ | □ | □ | □ | □ | □ |

| **20. Please indicate the extent to which you agree or disagree with the following statements by placing a tick (✓) in the appropriate box:** | | | | | | |
| --- | --- | --- | --- | --- | --- | --- |
|  | **Strongly agree** | **Agree** | **Neither agree nor disagree** | **Disagree** | **Strongly disagree** | **Don’t know** |
| It’s important for me to know a patient’s diagnosis/reason for admission to hospital | □ | □ | □ | □ | □ | □ |
| It’s important for me to know a patient’s diagnosis/reason for admission to intermediate care | □ | □ | □ | □ | □ | □ |
| It’s important for me to know the reason(s) for changes made to patients’ medication in hospital | □ | □ | □ | □ | □ | □ |
| It’s important for me to know the reason(s) for changes made to patients’ medication in intermediate care | □ | □ | □ | □ | □ | □ |
| I think community pharmacists should have electronic access to patients’ medical records in community pharmacies | □ | □ | □ | □ | □ | □ |
| I think patients should be registered with one community pharmacy to ensure continuity of care at healthcare interfaces | □ | □ | □ | □ | □ | □ |

Do you have any further comments on the communication of patients’ medication information when transferring between healthcare settings?

--------------------------------------------------------------------------------------------------------------------------------------------------------------------------------------------------------------------------------------------------------------------------------------------------------------------------------------------------------------------------------------------------------------------------------------------------------------------------------------------------------------------------------------------------------

­­­­

| **SECTION 4: COMMUNITY PHARMACISTS AND INTERMEDIATE CARE** |
| --- |

***The following questions concern your views on your level of confidence in your ability to provide services as a community pharmacist to patients and/or staff in intermediate care facilities, if the opportunity presented.***

**21. Please rate each of the following statements by circling the number that best applies to you for each statement:**

|  | **Could not do at all** | |  | |  | |  | | **Highly certain could do** | |
| --- | --- | --- | --- | --- | --- | --- | --- | --- | --- | --- |
| I could counsel intermediate care patients on their medications | 1 | 2 | 3 | 4 | 5 | 6 | 7 | 8 | 9 | 10 |
| I could provide education to intermediate care facility staff on medicines management | 1 | 2 | 3 | 4 | 5 | 6 | 7 | 8 | 9 | 10 |
| I could reconcile intermediate care patients’ medications | 1 | 2 | 3 | 4 | 5 | 6 | 7 | 8 | 9 | 10 |
| I could provide prescribing advice, including making recommendations to prescribers on the appropriateness of medications for individual patients in intermediate care | 1 | 2 | 3 | 4 | 5 | 6 | 7 | 8 | 9 | 10 |

**22. Many new services are subject to barriers or obstacles, which can hinder their implementation. Please consider the following potential barriers to a new community pharmacy service involving intermediate care and rate each in order of importance to you, where 1 is the most important barrier and 7 is the least important barrier *(please use each number only once).***

| **Barrier** | **Rating (1-7)** |
| --- | --- |
| Current workload (too busy to take on additional service) |  |
| Cover needed (*e.g.* locum/relief pharmacist) to facilitate participation in service |  |
| Expected provision of reimbursement for service |  |
| Level of clinical knowledge required to provide service |  |
| Working alongside GPs in intermediate care facilities |  |
| Working alongside intermediate care facility staff |  |
| IT resources |  |

**Do you have any further comments on the provision of services by community pharmacists to intermediate care patients and/or facilities?**

--------------------------------------------------------------------------------------------------------------------------------------------------------------------------------------------------------------------------------------------------------------------------------------------------------------------------------------------------------------------------------------------------------------------------------------------------------------------------------------------------------------------------------------------------------


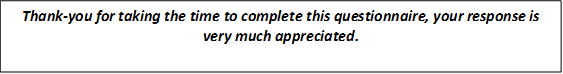

Supplement: Supplementary file 1 — Supplementary material 1 (DOCX 30 kb) [file 11096_2016_377_MOESM1_ESM.docx]
